# Supplementary material for: Developing a Machine Learning ‘Smart’ Polymerase Chain Reaction Thermocycler Part 2: Putting the Theoretical Framework into Practice
Source: Genes (Basel). 2024 Sep 12;15(9):1199. doi: 10.3390/genes15091199 (PMC11431514; doi:10.3390/genes15091199)

SUPPLEMENTARY MATERIALS:

a) One-step changes to annealing/extension timing

Table S1: Breakdown of average DNA profile quality of DNA profiles produced using various DNA amplification protocols with altered denaturation timing and annealing/extension timing stages. The protocol names are coloured based on the amplification kit used for each series where white with black text identifies the GlobalFiler and Investigator Quantiplex Pro Combination Kit and black with white text identifies GlobalFiler only. The profile quality scores are coloured based on a gradient where dark green is indicative of high-quality scores and dark red is indicative of low-quality scores within each time weighting bracket.

Comparing DNA Profile Quality Between Profiles Produced Using *Various* DNA Amplification Protocols

Using GlobalFiler & Investigator Quantiplex Pro Combination Kit and GlobalFiler Only with 0.5ng Human DNA

	Peak Height Statistics (RFU)			Penalties		Raw Profile Quality Score		Time Mildly Important		Time Highly Important	
	Mean	*Standard Deviation*	*COV*	*Peak Height*	*COV*	*Average Score*	*Range*	*Average Score*	*Range*	*Average Score*	*Range*
**GlobalFiler Validated Manufacturer's Protocol**											
Standard	4083	2573	0.63	2.54×10^{-4}	1.93×10^{-11}	-8.40	-8.06 to -8.67	-9.38	-9.04 to -9.65	-13.30	-12.96 to -13.57
Standard	1460	1017	0.71	1.40×10^{-6}	5.51×10^{-13}	-11.09	-10.32 to -12.00	-12.07	-11.30 to -12.98	-15.99	-15.22 to -16.90
**Altered Denaturation Timing Protocols**											
Bulk Change 1	2180	2254	1.03	1.16×10^{-5}	4.57×10^{-20}	-10.80	-10.17 to -11.41	-11.68	-11.05 to -12.29	-15.20	-14.57 to -15.81
Bulk Change 1	587	584	1.05	1.57×10^{-7}	2.44×10^{-19}	-13.36	-11.74 to -14.59	-14.24	-12.62 to -15.47	-17.76	-16.14 to -18.99
Bulk Change 2	692	981	1.42	4.05×10^{-8}	1.43×10^{-27}	-13.92	-13.47 to -14.22	-14.66	-14.21 to -14.96	-17.62	-17.17 to -17.92
Bulk Change 2	192	260	1.93	5.20×10^{-9}	5.35×10^{-13}	-15.93	-13.28 to -18.08	-16.67	-14.02 to -18.82	-19.63	-16.98 to -21.78
Gradient Change 1	3564	2800	0.78	1.61×10^{-4}	5.21×10^{-14}	-9.00	-8.67 to -9.33	-9.77	-9.44 to -10.10	-12.85	-12.52 to -13.18
Gradient Change 1	1851	1479	0.87	6.22×10^{-5}	6.00×10^{-14}	-11.25	-8.69 to -13.38	-12.02	-9.46 to -14.15	-15.10	-12.54 to -17.23
Gradient Change 2	884	1020	1.16	1.06×10^{-7}	9.50×10^{-23}	-13.01	-12.43 to -13.72	-13.70	-13.12 to -14.41	-16.46	-15.88 to -17.17
Gradient Change 2	301	365	1.43	1.33×10^{-8}	3.26×10^{-22}	-14.70	-13.03 to -16.13	-15.39	-13.72 to -16.82	-18.15	-16.48 to -19.58
**Altered Annealing/Extension Timing Protocols**											
Bulk Change 1	2917	1958	0.67	5.52×10^{-5}	2.38×10^{-11}	-9.32	-8.62 to -9.68	-9.86	-9.25 to -10.31	-12.38	-11.77 to -12.83
Bulk Change 1	683	722	1.14	1.60×10^{-7}	2.45×10^{-19}	-13.37	-11.30 to -14.80	-14.10	-11.93 to -15.43	-17.02	-14.45 to -17.95
Bulk Change 2	530	687	1.30	1.99×10^{-8}	7.97×10^{-25}	-13.96	-13.46 to -14.23	-14.44	-13.94 to -14.71	-16.36	-15.86 to -16.63
Bulk Change 2	174	511	3.13	3.96×10^{-9}	2.74×10^{-56}	-18.65	-17.25 to -19.89	-19.13	-17.73 to -20.37	-21.05	-19.65 to -22.29
Gradient Change 1	3578	2237	0.63	1.75×10^{-4}	2.53×10^{-11}	-8.72	-8.18 to -9.94	-9.44	-8.90 to -10.66	-12.32	-11.78 to -13.54
Gradient Change 1	1387	871	0.84	1.46×10^{-5}	5.98×10^{-11}	-11.73	-8.85 to -15.00	-12.45	-9.57 to -15.72	-15.33	-12.45 to -18.60
Gradient Change 2	3343	1977	0.60	1.38×10^{-4}	9.04×10^{-11}	-8.80	-8.24 to -9.98	-9.49	-8.93 to -10.67	-12.25	-11.69 to -13.43
Gradient Change 2	1011	809	0.84	1.01×10^{-6}	8.77×10^{-15}	-12.25	-10.31 to -13.59	-12.84	-11.00 to -14.28	-15.60	-13.76 to -17.04

Table S2: Breakdown of average standardised DNA profile quality of DNA profiles produced using various DNA amplification protocols with altered denaturation timing and annealing/extension timing stages. The protocol names are coloured based on the amplification kit used for each series where white with black text identifies the GlobalFiler and Investigator Quantiplex Pro Combination Kit and black with white text identifies GlobalFiler only. The standardised quality scores are coloured based on a gradient where dark green is indicative of high-quality scores and dark red is indicative of low-quality scores within each time weighting bracket.

Comparing DNA Profile Quality Between Profiles Produced Using *Various* DNA Amplification Protocols
Using GlobalFiler & Investigator Quantiplex Pro Combination Kit and GlobalFiler Only with 0.5ng Human DNA

	Peak Height Statistics (RFU)			Penalties		Standardised Raw Score		Standardised Time Mildly Important Score		Standardised Highly Important Score	
	Mean	*Standard Deviation*	*COV*	*Peak Height*	*COV*	*Average Score*	*Range*	*Average Score*	*Range*	*Average Score*	*Range*
**GlobalFiler Validated Manufacturer's Protocol**											
Standard	4083	2573	0.63	2.54×10^{-4}	1.93×10^{-11}	0.00	-0.27 to 0.33	0.00	-0.27 to 0.33	0.00	-0.27 to 0.33
Standard	1460	1017	0.71	1.40×10^{-6}	5.51×10^{-13}	0.00	-0.91 to -0.76	0.00	-0.91 to 0.76	0.00	-0.91 to 0.76
**Altered Denaturation Timing Protocols**											
Bulk Change 1	2180	2254	1.03	1.16×10^{-5}	4.57×10^{-20}	-2.40	-3.01 to -1.77	-2.30	-2.91 to -1.67	-1.90	-2.51 to -1.27
Bulk Change 1	587	584	1.05	1.57×10^{-7}	2.44×10^{-19}	-2.27	-2.82 to -0.65	-2.17	-3.41 to -0.55	-1.77	-3.01 to -0.15
Bulk Change 2	692	981	1.42	4.05×10^{-8}	1.43×10^{-27}	-5.52	-5.83 to -5.08	-5.28	-5.59 to -4.84	-4.32	-4.63 to -3.88
Bulk Change 2	192	260	1.93	5.20×10^{-9}	5.35×10^{-13}	-4.84	-6.99 to -2.19	-4.60	-6.75 to -1.95	-3.64	-5.79 to -0.99
Gradient Change 1	3564	2800	0.78	1.61×10^{-4}	5.21×10^{-14}	-0.52	-0.93 to -0.27	-0.31	-0.72 to -0.06	0.53	0.12 to 0.78
Gradient Change 1	1851	1479	0.87	6.22×10^{-5}	6.00×10^{-14}	-0.16	-2.30 to 2.40	0.05	-2.09 to 2.61	0.89	-1.25 to 3.45
Gradient Change 2	884	1020	1.16	1.06×10^{-7}	9.50×10^{-23}	-4.61	-5.32 to -4.75	-4.32	-5.03 to -3.74	-3.16	-3.87 to -2.59
Gradient Change 2	301	365	1.43	1.33×10^{-8}	3.26×10^{-22}	-3.62	-5.04 to -1.95	-3.33	-4.75 to -1.66	-2.17	-3.14 to -0.50
**Altered Annealing/Extension Timing Protocols**											
Bulk Change 1	2917	1958	0.67	5.52×10^{-5}	2.38×10^{-11}	-0.83	-1.28 to -0.22	-0.48	-0.93 to 0.13	0.92	0.47 to 1.53
Bulk Change 1	683	722	1.14	1.60×10^{-7}	2.45×10^{-19}	-2.10	-3.71 to -0.21	-1.75	-3.36 to 0.14	-0.35	-1.96 to 0.85
Bulk Change 2	530	687	1.30	1.99×10^{-8}	7.97×10^{-25}	-5.56	-5.83 to -5.06	-5.06	-5.33 to -4.56	-3.06	-3.33 to =2.56
Bulk Change 2	174	511	3.13	3.96×10^{-9}	2.74×10^{-56}	-7.56	-8.81 to -6.16	-7.06	-8.31 to -5.66	-5.06	-6.31 to -3.66
Gradient Change 1	3578	2237	0.63	1.75×10^{-4}	2.53×10^{-11}	-0.32	-1.54 to 0.22	-0.06	-1.28 to 0.48	0.98	-0.24 to 1.52
Gradient Change 1	1387	871	0.84	1.46×10^{-5}	5.98×10^{-11}	-0.64	-3.91 to 2.24	-0.38	-3.65 to 2.50	0.66	-2.61 to 2.85
Gradient Change 2	3343	1977	0.60	1.38×10^{-4}	9.04×10^{-11}	-0.40	-1.59 to 0.16	-0.11	-1.30 to 0.45	1.05	-0.14 to 1.61
Gradient Change 2	1011	809	0.84	1.01×10^{-6}	8.77×10^{-15}	-1.06	-2.50 to 0.78	-0.77	-2.08 to -1.29	0.39	-1.05 to 2.23

b) One-step changes to denaturation temperature

Table S3: Breakdown of average DNA profile quality of DNA profiles produced using various DNA amplification protocols with altered denaturation temperatures. The protocol names are coloured based on the amplification kit used for each series where white with black text identifies the GlobalFiler and Investigator Quantiplex Pro Combination Kit and black with white text identifies GlobalFiler only. The profile quality scores are coloured based on a gradient where dark green is indicative of high-quality scores and dark red is indicative of low-quality scores.

Comparing DNA Profile Quality Between Profiles Produced Using *Various* DNA Amplification Protocols

Using GlobalFiler & Investigator Quantiplex Pro Combination Kit and GlobalFiler Only with 0.5ng Human DNA

	Peak Height Statistics (RFU)			Penalties		Standardised Profile Quality Score		Raw Profile Quality Score	
	Mean	*Standard Deviation*	*COV*	*Peak Height*	*COV*	*Average*	*Range*	*Average*	*Range*
**GlobalFiler Validated Manufacturer's Protocol**									
Standard	4083	2573	0.63	2.54×10^{-4}	1.93×10^{-11}	0.00	-0.27 to 0.33	-8.40	-8.06 to -8.67
Standard	1460	1017	0.71	1.40×10^{-6}	5.51×10^{-13}	0.00	-0.91 to -0.76	-11.09	-10.32 to -12.00
**Altered Denaturation Temperature Protocols**									
Bulk Change 1	1233	1427	1.17	6.18×10^{-7}	5.68×10^{-23}	-4.04	-4.73 to -3.24	-12.44	-11.64 to -13.12
Bulk Change 1	788	876	1.09	8.36×10^{-8}	2.77×10^{-19}	-1.94	-2.29 to -1.71	-13.03	-12.58 to -13.38
Bulk Change 2	1078	1318	1.23	2.35×10^{-7}	1.41×10^{-24}	-4.41	-5.17 to -3.97	-12.81	-12.37 to -13.57
Bulk Change 2	398	495	1.26	1.13×10^{-8}	1.95×10^{-24}	-3.04	-3.67 to -2.72	-14.13	-13.81 to -14.78
Gradient Change 1	1136	1435	1.26	2.72×10^{-7}	8.46×10^{-25}	-4.38	-4.61 to -4.03	-12.78	-12.43 to -13.01
Gradient Change 1	1133	1099	1.03	7.95×10^{-7}	5.58×10^{-16}	-1.24	-2.61 to -0.06	-12.33	-11.03 to -13.70
Gradient Change 2	3124	2653	0.85	8.33×10^{-5}	7.42×10^{-16}	-1.06	-1.66 to -0.06	-9.46	-8.76 to -10.06
Gradient Change 2	890	787	1.03	1.36×10^{-6}	7.17×10^{-16}	-1.74	-3.48 to 0.80	-12.82	-10.29 to -14.57

c) Two-step changes

Table S4: Breakdown of average DNA profile quality of DNA profiles produced using various optimised PCR protocols. The protocol names are coloured based on the amplification kit used for each series where white with black text identifies the GlobalFiler and Investigator Quantiplex Pro Combination Kit and black with white text identifies GlobalFiler only. The profile quality scores are coloured based on a gradient where dark green is indicative of high-quality scores and dark red is indicative of low-quality scores.

Comparing DNA Profile Quality Between Profiles Produced Using *Various* Optimised DNA Amplification Protocols

Using GlobalFiler & Investigator Quantiplex Pro Combination Kit and GlobalFiler Only with 0.5ng Human DNA

	Peak Height Statistics (RFU)			Penalties		Raw Profile Quality Scores		Time Mildly Important		Time Highly Important	
	Mean	*Standard Deviation*	*COV*	*Peak Height*	*COV*	*Average Score*	*Range*	*Average Score*	*Range*	*Average Score*	*Range*
**GlobalFiler Validated Manufacturer's Protocol**											
Standard	4083	2573	0.63	2.54×10^{-4}	1.93×10^{-11}	-8.40	-8.06 to -8.67	-9.38	-9.04 to -9.65	-13.30	-12.96 to -13.57
Standard	1460	1017	0.71	1.40×10^{-6}	5.51×10^{-13}	-11.09	-10.32 to -12.00	-12.07	-11.30 to -12.98	-15.99	-15.22 to -16.90
**Optimised Protocol**											
Version 1	1946	1591	0.83	6.95×10^{-6}	2.32×10^{-15}	-10.67	-9.84 to -11.96	-11.37	-10.54 to -12.66	-14.17	-13.34 to -15.46
Version 1	444	495	1.17	1.43×10^{-8}	2.60×10^{-19}	-13.85	-13.01 to -15.05	-14.55	-13.71 to -15.75	-17.35	-16.51 to -18.55
Version 2	1911	1848	0.98	9.79×10^{-6}	9.83×10^{-19}	-11.08	-9.86 to -12.43	-11.78	-10.56 to -13.13	-14.58	-13.36 to -15.93
Version 2	207	297	1.49	4.28×10^{-9}	5.13×10^{-28}	-15.02	-14.56 to -15.90	-15.72	-15.26 to -16.60	-18.52	-18.06 to -19.40
Version 3	2672	1896	0.72	3.81×10^{-5}	5.05×10^{-13}	-9.59	-8.94 to -10.89	-10.27	-9.62 to -11.57	-12.99	-12.34 to -14.29
Version 3	452	472	1.05	1.44×10^{-8}	3.64×10^{-20}	-13.58	-13.28 to -13.97	-14.26	-13.96 to -14.65	-16.98	-16.68 to -17.73
Version 4	1845	1821	1.01	4.88×10^{-6}	1.96×10^{-18}	-11.20	-10.26 to -12.66	-11.87	-10.93 to -13.33	-14.55	-13.61 to -16.01
Version 4	189	302	1.70	4.24×10^{-9}	3.39×10^{-30}	-15.52	-14.56 to -16.71	-16.19	-15.23 to -17.38	-18.87	-17.91 to -20.06

Table S5: Breakdown of average standardised DNA profile quality of DNA profiles produced using various optimised PCR protocols. The protocol names are coloured based on the amplification kit used for each series where white with black text identifies the GlobalFiler and

Investigator Quantiplex Pro Combination Kit and black with white text identifies GlobalFiler only. The standardised profile quality scores are coloured based on a gradient where dark green is indicative of high-quality scores and dark red is indicative of low-quality scores.

Comparing DNA Profile Quality Between Profiles Produced Using *Various* Optimised DNA Amplification Protocols

Using GlobalFiler & Investigator Quantiplex Pro Combination Kit and GlobalFiler Only with 0.5ng Human DNA

	Peak Height Statistics (RFU)			Penalties		Standardised Raw Score		Standardised Time Mildly Important Score		Standardised Time Highly Important Score	
	Mean	*Standard Deviation*	*COV*	*Peak Height*	*COV*	*Average Score*	*Range*	*Average Score*	*Range*	*Average Score*	*Range*
**GlobalFiler Validated Manufacturer's Protocol**											
Standard	4083	2573	0.63	2.54×10^{-4}	1.93×10^{-11}	0.00	-0.27 to 0.33	0.00	-0.27 to 0.33	0.00	-0.27 to 0.33
Standard	1460	1017	0.71	1.40×10^{-6}	5.51×10^{-13}	0.00	-0.91 to -0.76	0.00	-0.91 to 0.76	0.00	-0.91 to 0.76
**Optimised Protocol**											
Version 1	1946	1591	0.83	6.95×10^{-6}	2.32×10^{-15}	-2.28	-3.56 to -1.44	-2.00	-3.28 to -1.16	-0.88	-2.16 to -0.04
Version 1	444	495	1.17	1.43×10^{-8}	2.60×10^{-19}	-2.76	-3.96 to -1.92	-2.48	-3.68 to -1.64	-1.36	-2.56 to -0.52
Version 2	1911	1848	0.98	9.79×10^{-6}	9.83×10^{-19}	-2.68	-4.04 to 1.46	-2.40	-3.76 to -1.18	-1.28	-2.64 to -0.06
Version 2	207	297	1.49	4.28×10^{-9}	5.13×10^{-28}	-3.93	-4.81 to -3.47	-3.65	-4.53 to -3.19	-2.53	-3.41 to -2.07
Version 3	2672	1896	0.72	3.81×10^{-5}	5.05×10^{-13}	-1.20	-2.49 to -0.54	-0.90	-2.19 to -0.24	0.30	-0.99 to 0.96
Version 3	452	472	1.05	1.44×10^{-8}	3.64×10^{-20}	-2.49	-2.88 to -2.20	-2.19	-2.58 to -1.90	-0.99	-1.38 to -0.70
Version 4	1845	1821	1.01	4.88×10^{-6}	1.96×10^{-18}	-2.80	-4.26 to -1.86	-2.49	-3.95 to -1.55	-1.25	-2.71 to -0.31
Version 4	189	302	1.70	4.24×10^{-9}	3.39×10^{-30}	-4.43	-5.62 to -3.48	-4.12	-5.31 to -3.17	-2.88	-4.07 to -1.93

STRmix settings used to analyse GlobalFiler DNA profile data


```xml
<?xml version="1.0" encoding="UTF-8" standalone="yes"?>
<profilingKit kitName="GlobalFiler_3500">
  <kitType>GlobalFiler</kitType>
  <sizeRegressionFile>GlobalFiler_SizeRegression.csv</sizeRegressionFile>
  <loci count="24">
    <locus repeatLength="4">D3S1358</locus>
    <locus repeatLength="4">vWA</locus>
    <locus repeatLength="4">D16S539</locus>
    <locus repeatLength="4">CSF1PO</locus>
    <locus repeatLength="4">TPOX</locus>
    <locus repeatLength="4">Yindel</locus>
    <locus>AMEL</locus>
    <locus repeatLength="4">D8S1179</locus>
    <locus repeatLength="4">D21S11</locus>
    <locus repeatLength="4">D18S51</locus>
    <locus repeatLength="4">DYS391</locus>
    <locus repeatLength="4">D2S441</locus>
    <locus repeatLength="4">D19S433</locus>
    <locus repeatLength="4">TH01</locus>
    <locus repeatLength="4">FGA</locus>
    <locus repeatLength="3">D22S1045</locus>
    <locus repeatLength="4">D5S818</locus>
    <locus repeatLength="4">D13S317</locus>
    <locus repeatLength="4">D7S820</locus>
    <locus repeatLength="4">SE33</locus>
    <locus repeatLength="4">D10S1248</locus>
    <locus repeatLength="4">D1S1656</locus>
    <locus repeatLength="4">D12S391</locus>
    <locus repeatLength="4">D2S1338</locus>
  </loci>
  <genderLoci count="1">
    <locus>AMEL</locus>
  </genderLoci>
  <kitSettings>
    <detectionThresholds>
      <threshold locus="D3S1358">75</threshold>
      <threshold locus="vWA">75</threshold>
      <threshold locus="D16S539">75</threshold>
      <threshold locus="CSF1PO">75</threshold>
      <threshold locus="TPOX">75</threshold>
      <threshold locus="Yindel">75</threshold>
      <threshold locus="D8S1179">75</threshold>
      <threshold locus="D21S11">75</threshold>
      <threshold locus="D18S51">75</threshold>
      <threshold locus="DYS391">75</threshold>
      <threshold locus="D2S441">75</threshold>
      <threshold locus="D19S433">75</threshold>
      <threshold locus="TH01">75</threshold>
      <threshold locus="FGA">75</threshold>
      <threshold locus="D22S1045">75</threshold>
      <threshold locus="D5S818">75</threshold>
      <threshold locus="D13S317">75</threshold>
      <threshold locus="D7S820">75</threshold>
      <threshold locus="SE33">75</threshold>
      <threshold locus="D10S1248">75</threshold>
      <threshold locus="D1S1656">75</threshold>
      <threshold locus="D12S391">75</threshold>
      <threshold locus="D2S1338">75</threshold>
    </detectionThresholds>
    <ignoredLoci>
      <locus>Yindel</locus>
      <locus>DYS391</locus>
    </ignoredLoci>
    <saturation>30000</saturation>
    <degradationStartsAt>-1.0</degradationStartsAt>
    <degradationMax>0.01</degradationMax>
    <dropinCap>100</dropinCap>
    <dropinRateParameter>1.0E-4</dropinRateParameter>
    <dropinGammaParameters>0.0,0.0</dropinGammaParameters>
    <allelicVariance>4.865,3.101</allelicVariance>
    <minVarFactor>0.5</minVarFactor>
    <locusAmpVariance>0.0217</locusAmpVariance>
```

<varianceMinimizationParameter>1000.0</varianceMinimizationParameter>
<stutters count="2">
<stutter name="Back Stutter">
<enabled>Y</enabled>
<positionRelativeToParent>-1,0</positionRelativeToParent>
<inverselyProportionalToParent>Y</inverselyProportionalToParent>
<stutterMax>0.3</stutterMax>
<stutterVariance>3.499,4.803</stutterVariance>
<regressionFile>GlobalFiler_Stutter_3500.txt</regressionFile>
<exceptionsFile>GlobalFiler_Stutter_Exceptions_3500.csv</exceptionsFile>
<applicableLoci all="true"/>
</stutter>
<stutter name="Forward Stutter">
<enabled>Y</enabled>
<positionRelativeToParent>1,0</positionRelativeToParent>
<inverselyProportionalToParent>N</inverselyProportionalToParent>
<stutterMax>0.15</stutterMax>
<stutterVariance>4.865,3.101</stutterVariance>
<regressionFile>GlobalFiler_Forward_Stutter_3500.txt</regressionFile>
<exceptionsFile></exceptionsFile>
<applicableLoci all="true"/>
</stutter>
</stutters>
</kitSettings>
</profilingKit>

DNA profile obtained from combined GlobalFiler/Quantiplex system amplifying 500pg DNA

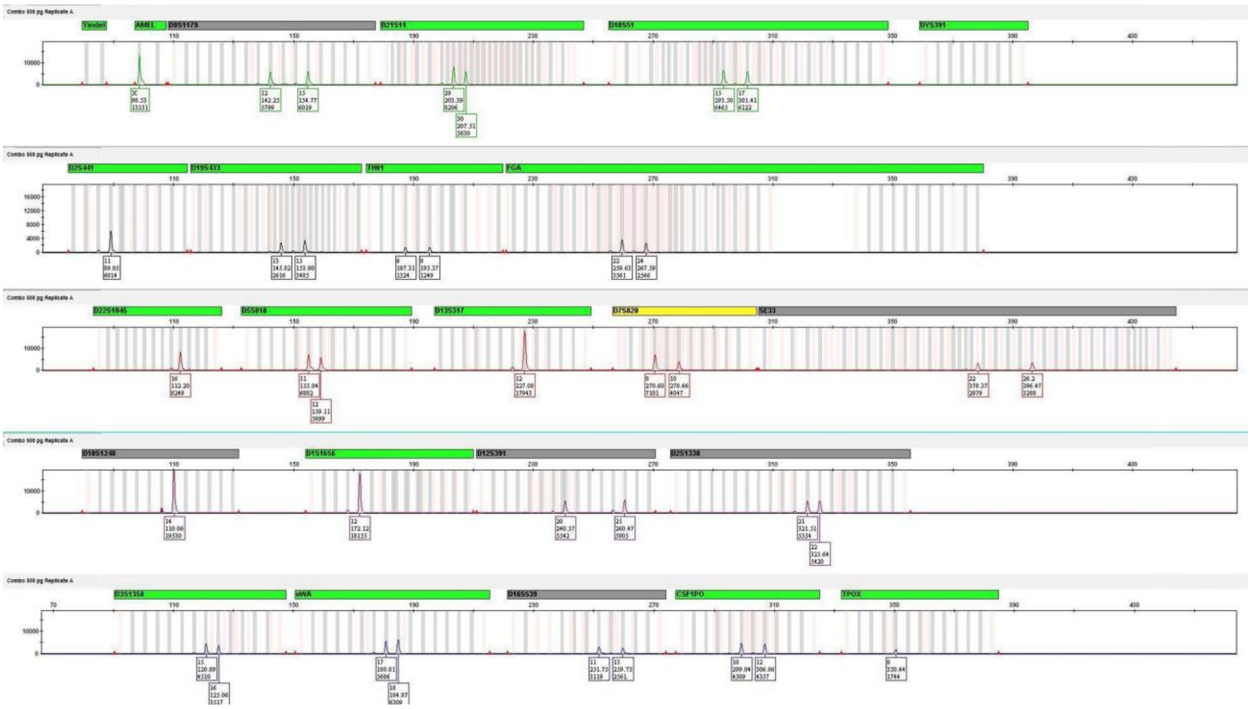

Supplement: Supplementary file 1 [file genes-15-01199-s001.zip › genes-3180230-supplementary.pdf]
